# Low-Carbon Watershed Management: Potential of Greenhouse Gas Reductions from Wastewater Treatment in Rural Vietnam

**DOI:** 10.1155/2016/6523217

**Published:** 2016-09-08

**Authors:** Lan Huong Nguyen, Geetha Mohan, Pu Jian, Kazuhiko Takemoto, Kensuke Fukushi

**Affiliations:** ^1^Graduate Program in Sustainability Science-Global Leadership Initiative (GPSS-GLI), Graduate School of Frontier Sciences, The University of Tokyo, Chiba 277-8563, Japan; ^2^Integrated Research System for Sustainability Science (IR3S), The University of Tokyo, Tokyo 113-8654, Japan; ^3^The United Nations University Institute for the Advanced Study of Sustainability, Tokyo 150-8925, Japan; ^4^The University of Tokyo Institutes for Advanced Studies (UTIAS), Integrated Research System for Sustainability Science (IR3S), The University of Tokyo, Tokyo 113-8654, Japan

## Abstract

Currently in many cities and rural areas of Vietnam, wastewater is discharged to the environment without any treatment, which emits considerable amount of greenhouse gas (GHG), particularly methane. In this study, four GHG emission scenarios were examined, as well as the baseline scenario, in order to verify the potential of GHG reduction from domestic wastewater with adequate treatment facilities. The ArcGIS and ArcHydro tools were employed to visualize and analyze GHG emissions resulting from discharge of untreated wastewater, in rural areas of Vu Gia Thu Bon river basin, Vietnam. By applying the current IPCC guidelines for GHG emissions, we found that a reduction of GHG emissions can be achieved through treatment of domestic wastewater in the studied area. Compared with baseline scenario, a maximum 16% of total GHG emissions can be reduced, in which 30% of households existing latrines are substituted by Japanese Johkasou technology and other 20% of domestic wastewater is treated by conventional activated sludge.

## 1. Introduction

Vietnam in recent years, after economic reforms (Doi Moi, 1986), has been experiencing rapid economic growth, with an average annual growth rate of 5.4% in gross domestic product (GDP). The GDP per capita in Vietnam is projected to reach around 2333 USD in the year 2030, which might bring an increase in energy consumption and impel a corresponding increase in GHG emissions. The energy sector is the largest source of Vietnam GHG emissions. In 2000, GHG emissions proportion from energy sector is 35%, equivalent to 52.8 TgCO_2_eq of total 150.9 TgCO_2_eq emissions [1], and will continue to increase as projected until 2030. Total emissions from energy, agriculture, land use, land use change, and forestry sectors are projected to be 169.2, 300.4, and 515.8 TgCO_2_eq in 2010, 2020, and 2030, respectively. Energy sector accounts for 91.3% of projected total emissions for 2030.

The waste and wastewater sector has a small share in total GHG emissions of the country. However, this sector emission is likely to increase, due to population growth and the lack of wastewater treatment in cities and as well in rural areas. The major GHGs including CH_4_ and N_2_O can be produced and emitted at many stages from sources and final disposal along the discharge pathways [2]. Currently, the urban population is 28%, and accessing of drainage and wastewater treatment facilities is still too low compared to accessing of drinking water supply services. There is only 40 to 50% of urban population served by sewage system, in which only 10% of the wastewater collected is treated with adequate treatment technology before entering the environment [3]. The rest of urban wastewater is discharged into natural water bodies. Under tropical climate with relatively high temperature throughout the year, anaerobic processes take place to different extents and methane is released to the atmosphere [4]. Meanwhile in rural areas, the most common sanitation practice is household latrines. Household wastewater (including gray water and black water) is collected in underground chambers and seepage to ground or discharged directly to farm land or gardens, eventually entering open water bodies. The high methane conversion factor (MCF) value of household latrines [4, 5] indicates a significant amount of methane emission from this treatment facility. Thus, having a proper wastewater treatment facility would reduce GHG emissions from domestic wastewater discharge and handling. However, the inventory of GHG emission from wastewater sector as well as options to reduce GHG emission in the sector was not described in any of climate change mitigation policies of the country. Also, taking the sectoral approach by targeting each sector (i.e., emissions targeted in VGGS [6]) in the economy might result in unbalance of development in the sector because many of the sectors are highly independent on each other especially the nexus of water and energy sector.

Methane emission from wastewater treatment is among the major sources of GHG emissions besides CO_2_ and N_2_O. Methane emission results from the two main routes: the decomposition of degradable organic under anaerobic condition and the air exposure of flow containing dissolved CH_4_. Under anaerobic condition, organic matters contained in wastewater are decomposed by anaerobic bacteria (e.g., the denitrification or dephosphorization process) resulting in the generation of CH_4_. Also, CH_4_ dissolved in wastewater could be released into atmosphere via surface exposure. Recent research on GHG emissions from domestic wastewater was seen mostly in small scale or some specific treatment processes. Work conducted by Ma et al. [7] is among few studies that focus on the general condition of domestic wastewater using the IPCC methods for GHG emissions inventory for wastewater discharge and handling (hereafter called IPCC method). This study found that the emissions from domestic wastewater treatment could be reduced by 10% or 0.0763 Mt in the year 2020 scenarios. However this study as well as IPCC method is significant regarding trend and peak of GHG emissions; the allocation of GHG from spatial dimension was not considered especially from emissions from the watershed. In a study by Galloway et al. [8], emission from watershed was analyzed because watersheds have structural and functional characteristics that can reflect and influence how humans interact within the watershed system. In this analysis, the watershed is considered as one of the four major reservoirs, namely, atmosphere, watershed, coastal and shelf region, and open oceans. The result of this study shows the major source of N is from N-fertilizer input. However, this study did not include inputs from sewage discharge to the watershed. Another related study from [9] examined the life cycle GHG emissions from sewage sludge treatment in the Tai Lake Watershed in China. This study selects different sludge treatment and disposal processes to reduce emissions in the watershed and suggests the optimum choice for the Tai Lake Watershed. The proportion of GHG emission from this process calculates about 40% of total emission from sewage treatment and handling [10, 11]. The case of Tai Lake is much different from other developing countries, where sewage discharged without proper treatment and became the major source of GHG emissions from rivers and streams. Therefore, it is important to study the GHG emissions from wastewater discharge and handling in the watershed to provide the optimum solution of the wastewater system for the river basin.

This paper describes briefly the status of water and wastewater sector in Vietnam followed by the estimation of GHG reduction from domestic wastewater by providing adequate wastewater treatment facilities under different emission scenarios for the Vu Gia Thu Bon river basin. GHG reductions from domestic wastewater handling and disposal from subwatershed are then analyzed using ArcGIS. Finally this paper addresses uncertainties in the calculation methods and then discusses the economical aspect of the proposed scenarios.

### 1.1. Vietnam Water and Wastewater Sector

Vietnam has relatively abundant water resources including surface and ground water existing in rivers, lakes, artificial reservoirs, and underground aquifers. Its dense river network consists of 2,372 rivers which are over 10 kilometers long and includes 109 main rivers with the total area of river basins 1,167,000 km^2^. The total annual river flow volume is approximately 847 billion m^3^. There are many natural lakes, ponds, lagoons, and pools, which are not sufficiently identified. It is estimated that the total area of ponds and lakes is merely 1,500 km^2^. Groundwater resource is recognized as significant reserve with estimated volume of 48 billion m^3^ per year.

With increasing economic growth in Vietnam in recent decades, declining water quality in the basins is becoming an acute problem. Urban areas are subject to serious degradation in water quality, whilst rural area faces problem of clean water access. With the rapid economic development, it is estimated that the urban population will double, which poses high pollution risk to the water resources. Sustainable development has to ensure water quality and environmental protection that would minimize the environmental impact. Domestic wastewater treatment is one of the first priorities; however, the lack of investment and payment for wastewater services poses challenges for the implementation of the system [3]. In addition, water is an energy intensive sector, which requires a large consumption of electricity for treatment of drinking water, wastewater, and water transportation. A number of studies in different countries and cities like India [12], China [13], Japan [14], Australia [15], and USA [16] have shown that energy consumption for water and wastewater treatment increases with higher GDP. In urban areas, energy consumption in water and wastewater sector is relatively higher than the country average. For instance, in California City, the water sector consumes 13% of the total electricity consumption of the city. The city Tokyo exhibits the similar case with an energy consumption of 903 kWh/cap/yr. This trend clearly indicates that the energy consumption for water and wastewater treatment increases with higher GDP relatively higher than the country average.

Vietnam has successfully progressed in improving its water supply situation over the past decades. The proportion of population using an improved drinking water source is increased by 28% from 1990 to 2010 [17]. According to Ministry of Construction [3, 18], until the year 2010, 18.15 million people could have access to drinking water, accounting for 69% of the total urban population, with an average water amount of 80–90 L/person/d in urban and 120–130 L/person/d in large cities. Assuming it takes about 0.3 kWh energy for producing 1 m^3^ clean water from surface water and 0.6 kWh for reclaimed wastewater, the amount of energy consumption for treatment of water supply is approximately 800 MWh/day and 1,600 MWh/day to treat wastewater generated from the urban population. Currently in many cities and rural areas of Vietnam, wastewater is not treated and discharged to the environment, which emits considerable amount of methane. In addition, wastewater discharge and wastewater treatment facilities produce the major greenhouse gases, carbon dioxide (CO_2_), methane (CH_4_), and nitrous oxide (N_2_O). Therefore, sustainable wastewater treatment technology has to consider the balance of energy input as well as emission from water treatment process.

## 2. Materials and Methods

### 2.1. Study Area

**Figure 1:**
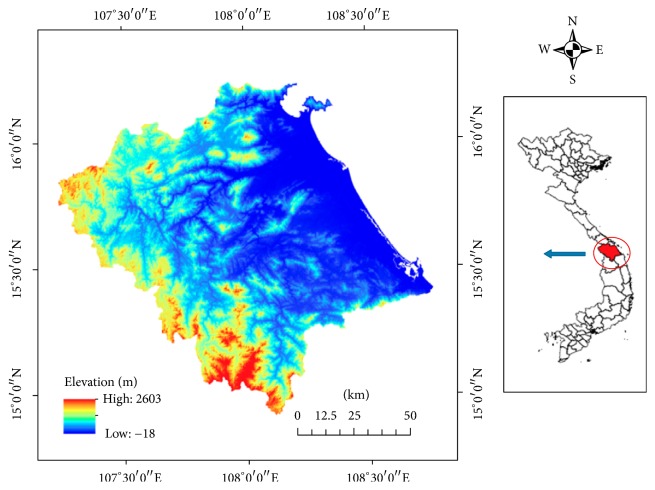
Vu Gia Thu Bon river basin in central Vietnam.

The study site locates at the Vu Gia Thu Bon river basin, the most important social-economic hub of the Central Vietnam. The river system originates from mountains of the Kon Tum province and flows through Quang Nam province and Da Nang city before discharging to the sea. Two main rivers from the system named Vu Gia and Thu Bon both originates from the Truong Son mountain range (Figure 1). This river basin is the major water resource for the region especially of high potential for hydropower development. The area with 75% of hills and mountains is favored for small and medium water resources development project including small hydropower and decentralized water resource management.

The economic development in this region is however very low, compared to the other river basins such as Red River basin in the north and Dong Nai river basin in the south. Quang Nam province covers the largest areas of the three main provinces in the Vu Gia Thu Bon river basin. As shown in Table 1, Quang Nam has a relatively low GDP per capita compared to the national average with 1,248 US dollars [20]. The ethnic minorities that have a large population living below the poverty line inhabit the mountainous districts. Lack of electricity for development as well as water supply and sanitation has been seen as the most vulnerable issue of this region.

**Figure 2:**
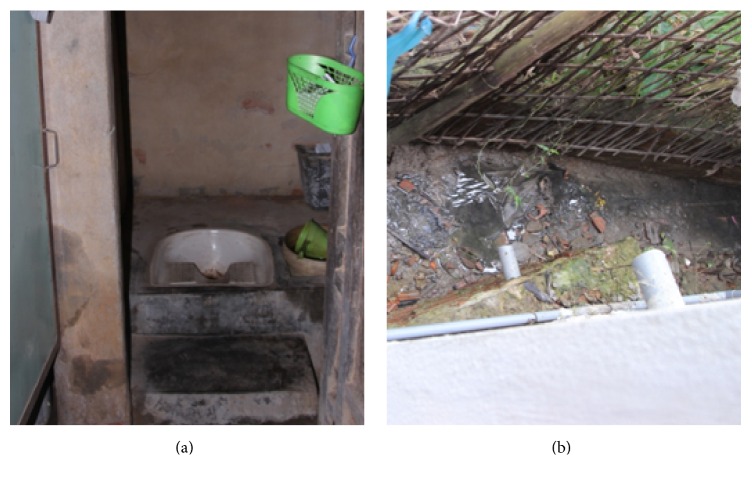
Typical household pour-flush pit latrine (a). Black water overflow and gray water discharged to backyard (b) (photo taken by author).

Household pour-flush pit latrines are seen as the most common wastewater treatment facility in this region, which is similar to other parts of the country. Rural households usually install underground chambers to collect wastewater mostly black water. Generally, black water is collected and seepage into the ground, sludge, and feces is settled while gray water is discharged to gardens or farmland (Figure 2). In some cases especially in towns, or wealthy households, wastewater collected in septic tanks is mixture of black and gray water or if there is presence of sewer, septic tanks only receive black water. In addition due to lack of sludge treatment infrastructure and service, sludge is not emptied regularly. The house owners usually empty septic tanks when they are full which can take from 1 to 10 years [25]. There are several micro credit facilities to rural household to borrow a certain amount of cash average 600 US dollars, to construct latrine with zero percentage of interest rate from the local government funds.

### 2.2. Methodology

#### 2.2.1. Watershed Delineation

**Figure 3:**
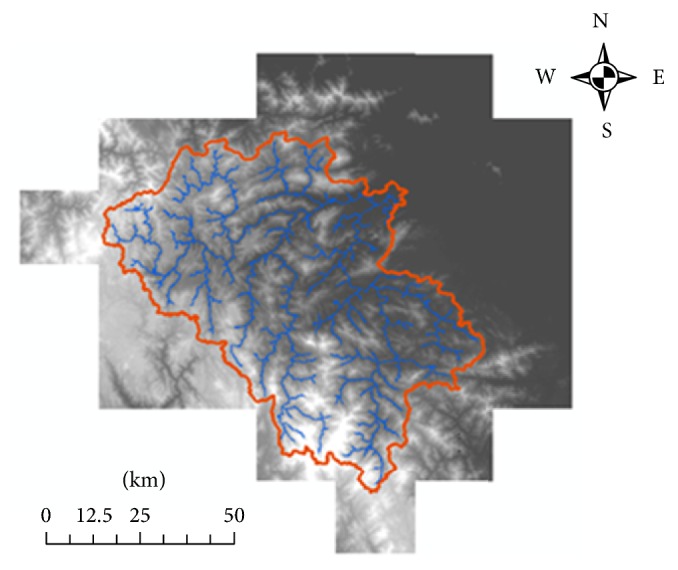
Watershed delineation using ArcHydro 9.1.

Two digital maps, DEM, and land use in raster format are needed to derive watershed characteristics. DEM resolution of 30 m is derived from digital topographic map, scale 1 : 50000. Both land use and topographic map are obtained from Quang Nam Natural Resource and Environment Department. Watershed boundaries are extracted from DEM using ArcHydro (Figure 3). The study area boundaries were identified that cover 103 watersheds with total area of 2,924 km^2^.

**Figure 4:**
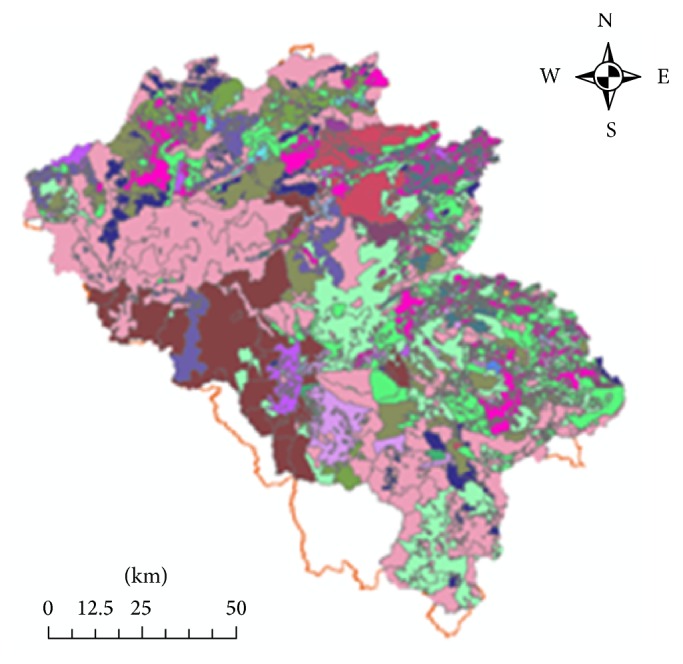
Land use map.

**Figure 5:**
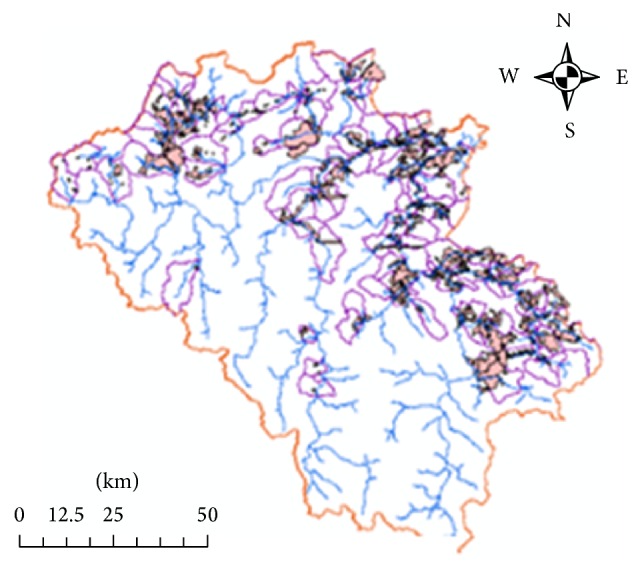
Rural settlement designated in watersheds.

**Figure 6:**
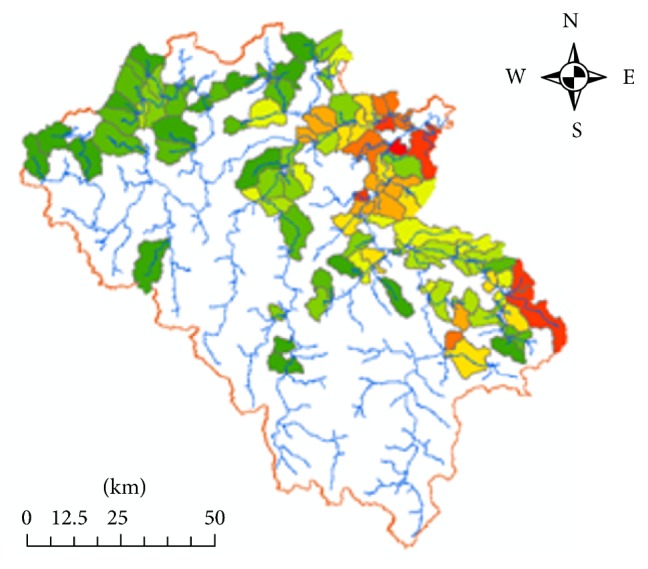
Population density (2012).

The land use map (Figure 4) and subwatershed map are integrated so that the residential area is covered within the watershed boundaries (Figure 5). Population was obtained at communal level from Quang Nam Statistical Office, and then total population living in designated residential area in each of the watersheds was calculated (Figure 6).

#### 2.2.2. Methane Emission Analysis from Domestic Wastewater Treatment and Discharge

**Figure 7:**
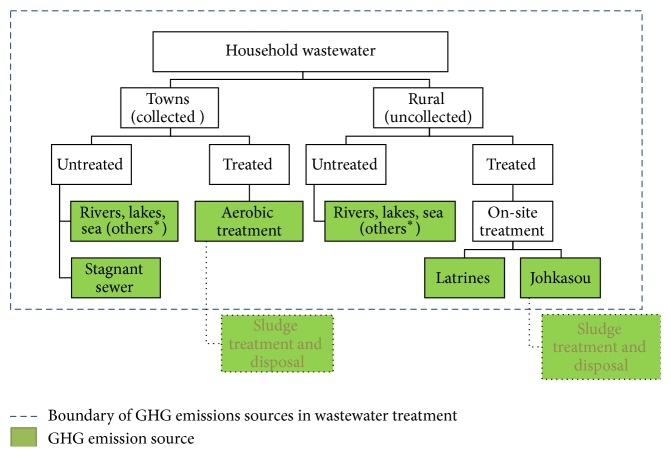
System boundary and wastewater treatment pathways. ∗ refers to the term "others" as indicated in Table 3

GHG emissions from watersheds are calculated based on GHG protocol and IPCC Guidelines for National Greenhouse Gas Inventories [4]. The boundary of this study includes direct GHG emissions, that is, methane emission from domestic wastewater treatment and indirect GHG emissions through electricity import for system operation (Figure 7). In scope of this study, CO_2_ and N_2_O emissions from wastewater treatment are not considered. The CO_2_ emission is biogenic origin that is part of natural carbon cycle and does not contribute to global warming. Therefore it is not included in natural GHG emissions inventory. The N_2_O emission is not included because of insufficient data and large uncertainties associated with the IPCC default emission factors for N_2_O from effluent [4]. The system boundary and wastewater discharge pathways are shown in Figure 7.

The total CH_4_ emission from domestic wastewater discharged in watershed is calculated based on the following equations.

Total CH_4_ emission from domestic wastewater discharged in watershed *i* is

(1) $$\begin{array}{c} {CH}_{4i}=\left[\;\underset{k,j}{\sum }\left(\;U_{k}\times T_{k,j}{EF}_{j}\;\right)\;\right]\left(\;{TOW}_{i}-S_{i}\;\right)-R_{i}, \end{array}$$

where  CH_4*i*_ is CH_4_ emission in inventory year in watershed *i*, kg CH_4_/year, EF_*j*_ is emission factor of treatment pathway *j*, kg CH_4_/kg BOD (Section 2.3.2), TOW_*i*_ is total organically degradable carbon in domestic wastewater discharged in watershed *i*, *S*_*i*_ is organic component removed as sludge in inventory year, kg BOD/year, *U*_*k*_ is fraction of population in income group *k* in inventory year, *U*_*k*_ = 1, *T*_*k*,*j*_ is degree of utilization of treatment pathway *j*, and *R*_*i*_ is amount of CH_4_ recovered in inventory year, kg CH_4_/year.

There are several assumptions to the calculation of total methane emission as follows:

 Fraction of degradable organic component removed as sludge *S*_*i*_ = 0.
 No recovery or flaring of methane *R*_*i*_ = 0.

### 2.3. Data

#### 2.3.1. Activity Data

We used IPCC default value for the amount of organics content of domestic wastewater. Because the value of BOD in domestic wastewater depends on the wastewater treatment system, the quantity of discharged wastewater, and the degree of utilization of the wastewater treatment, the lower value of 40 g/person/day was chosen for the analysis. Also the gross domestic product (GDP) is an important indicator for the economic development of the region, the increasing of GDP in economic development impels the increasing of BOD in domestic wastewater. Thus, according to expert judgment, the BOD value project for the next 30 years was assumed as 60 g/person/day.

#### 2.3.2. Wastewater Treatment Pathways and Emission Factors

The treatment options included in the analysis cover household latrines, onsite advanced treatment Johkasou, and centralized conventional aerobic treatment. The emission factor from different treatment pathways is calculated as follows:

(2) $$\begin{array}{c} EF_{j}=B_{o}\times {MCF}_{j}, \end{array}$$

where EF_*j*_ is the emission factor of treatment pathway *j*, kg CH_4_/kg BOD, and *B*_*o*_ is the maximum CH_4_ producing capacity, kg CH_4_/kg BOD. The IPCC default value is 0.6 kg CH_4_/kg BOD and MCF_*j*_ is the methane correction factor of treatment pathway.

The methane correction factors for household latrines, sewer, and centralized aerobic treatment were selected from the IPCC guidelines. The value was 0.7 for household latrines, 0.5 for sewer system, and 0.2 for conventional activated sludge treatment.

In the project scenarios of low population density especially in the upstream of the basin, the Japan decentralize treatment system (Johkasou) was selected. The Johkasou unit can perform tertiary treatment to ensure the effluent BOD below 20 mg/L. Originated in Japan, until the year 2010, the Johkasou system has been serving approximately 30 million people with 9% of total population [27]. The results of Johkasou treatment performance were shown in Table 2. These results were obtained from the standardized Johkasou with the capacity of 1 to 50 m^3^/day. The influent was the domestic wastewater with similar characteristic to the household wastewater in the studied region.

The emission calculated for Johkasou system includes methane and indirect emission from electricity consumption. Emission factor used was 1.84 kgCH_4_/person/year [28] and energy consumption was 101 W for the ten-person Johkasou unit [29]. Grid emission factor for Vietnam grid was 0.56 tCO_2_/MWh and was expected to increase to 0.72 tCO_2_/MWh in year 2030 [30].

#### 2.3.3. Scenario Description

The model simulates carbon emissions for 30 years from January 2012 to December 2041 under four scenarios. Scenarios description is provided in Table 3.

According to population projection in the region until 2050, population growth rate is assumed to be at 0.5% for all scenarios. In addition, due to GDP increasing and lifestyle improvement in the region, BOD value in domestic wastewater increases from 40 to 60 g/cap/day. Upon baseline scenario, four mitigation options to reduce GHG emissions from wastewater discharge and treatment are proposed, namely, scenario 1 to scenario 4. In relation to climate change scenarios developed for Vietnam, scenarios 1 and 2 represent the medium emission scenario (B2 (IPCC Fourth Assessment Report, B2 family scenario: continuously increasing population, but at a rate lower than A2; the emphasis being on local rather global solutions to economic, social, and environmental sustainability; intermediate levels of economic development; less rapid and more diverse technological change than in B1 family (medium emission scenario).)), while scenarios 3 and 4 represent the low emission scenario (B1 (IPCC Fourth Assessment Report, B1 family scenario: rapid economic growth, but with rapid changes towards a service and information economy; global population reaching the peak in 2050 and declining thereafter; reductions in material intensity and the introduction of clean and resource-efficient technologies; the emphasis on global solutions to economic, social, and environmental sustainability (low emission scenario).)).

In the baseline scenario set until 2041, the proportion of rural population having access to hygienic latrine is 80%; there are no wastewater sewers to collect wastewater. The major source of methane emission is from wastewater treated in household latrines. In scenario 1, twenty percent of rural population has access to hygienic latrine, 10% wastewater is collected by sewer, and 50% is treated with conventional aerobic treatment. Scenarios 2 and 4 exhibit that the Japanese Johkasou wastewater treatment could be introduced and served for 30% of the rural households by the year 2041. The proportion of rural population having access to hygienic latrine is 20%. The distributions of 10% and 20% wastewater of the rural population wastewater are collected by sewer and treated with aerobic treatment, respectively. Scenario 3 assumes that 50% of domestic wastewater discharged from total population will be treated in Johkasou system.

Electricity consumption for wastewater treatment with conventional activated sludge is 30 kWh/cap/year while energy consumption for Johkasou is 88 kWh/capita/year. In scenarios 3 and 4, energy consumption in Johkasou is reduced to 75 kWh/capita/year, and this reduction results from technology innovation and introducing of low energy Johkasou. The electricity source is from national grid with emission factor of 0.56 to 0.72 tCO_2_/MWh in years 2012 and 2041, respectively, except that in scenario 4 emission factor is 0.66 tCO_2_/MWh in year 2041. The grid emission reduction resulted from effectively implemented Green Growth Strategy in the power sector, with 8% reduction compared with baseline projection.

## 3. Results and Discussions

### 3.1. Methane Emission from Domestic Wastewater and Discharge: Baseline and Projection

A total number of 103 subwatersheds are studied for GHG emissions from domestic wastewater discharged by rural population living within the Vu Gia Thu Bon river basin. The area of watersheds ranges from 2.4 to 93.2 km^2^.  Table 4 shows ten watersheds with the highest GHG emissions, in a period of 30 years. Even though these watersheds represent only 17% of the total area, they attribute to 49% of total GHG emissions from wastewater.

**Figure 8:**
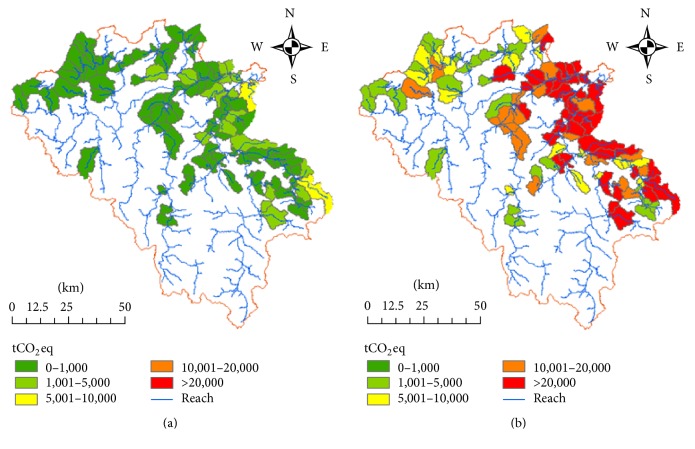
GHG emissions from domestic wastewater. (a) Inventory year 2012. (b) Baseline scenario for period of 30 years.

*Baseline Scenario*. As shown in Figure 8(b) and Table 5, based on the emission model output, total GHG emissions from wastewater in the area are 2.7 million tCO_2_eq for the 30-year project. An emission from wastewater per capita per year is 2.7% of total GHG emissions projection per capita in 2030 which is 5.0 tCO_2_eq (Vietnam's Second National Communication to UNFCCC, 2010).

**Figure 9:**
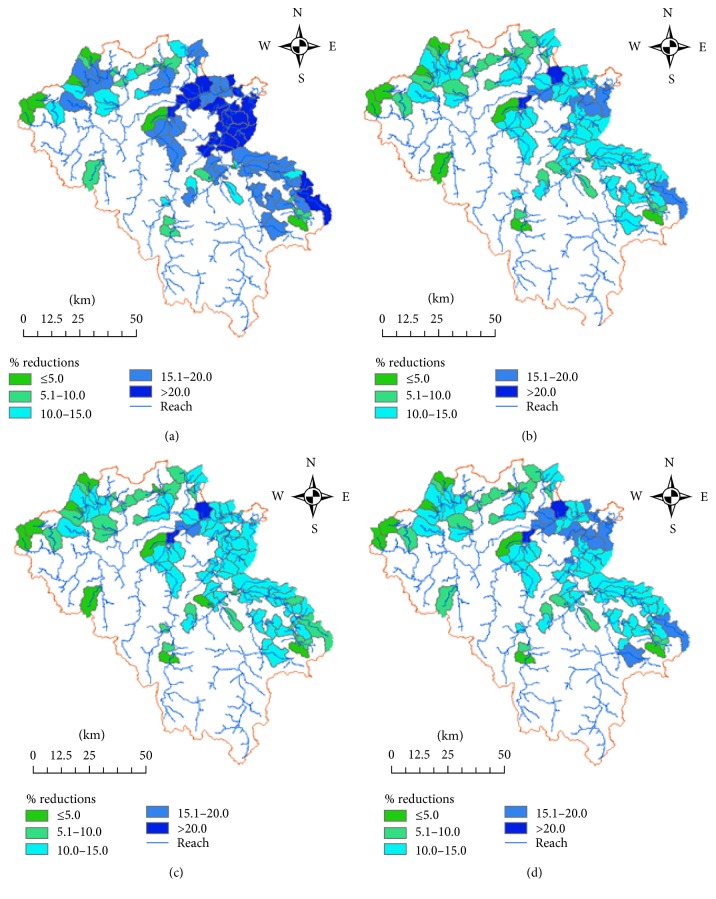
GHG emissions reduction with baseline (in percentage) from domestic wastewater treatment. (a) Scenario 1. (b) Scenario 2. (c) Scenario 3. (d) Scenario 4.

*Project Scenario 1*.  Figure 9(a) and Table 5 show that, by 2041, the major source of GHG emissions is from wastewater treated in household latrines, with some proportion of methane gas emitted from the sewer. Total emissions from wastewater in the area are 2.17 million tCO_2_eq for the total 30 years' simulation. The emissions from wastewater per capita per year are 0.11 tCO_2_eq. By treatment of wastewater, the total methane emission reduction is 0.59 million tCO_2_eq, which is 21% reduction compared with the baseline scenario.

*Project Scenario 2*.  Figure 9(b) and Table 5 indicate that sources of GHG emissions are from wastewater treated in household latrines, with GHG emitted from the sewer and from Johkasou treatment system. Total emissions from wastewater in the area are 2.33 million tCO_2_eq for the total 30 years' simulation. Emissions from wastewater per capita per year are 2.3% of total GHG emissions per capita in 2030 and the GHG emissions reduction for this project scenario is 12% compared with baseline scenario.

*Project Scenario 3*.  Figure 9(c) and Table 5 show that the total amount of GHG emissions from wastewater in the area for the period of 30 years is 2.42 million tCO_2_eq. Compared with baseline scenario, emissions reduction for 30-year simulation period is 0.33 million tCO_2_eq which accounted for 12%.

*Project Scenario 4*.  Figure 9(d) and Table 5 indicate that the major source of GHG emissions is from wastewater treated in household latrines, from sewer, and from Johkasou treatment system. The emission model generates total amount of methane emissions from wastewater in the area for period of 30 years being 2.31 million tCO_2_eq. Compared with baseline scenario, emissions reduction for project period is 0.45 million tCO_2_eq and the GHG emissions reduction per capita per year from wastewater is 0.12 tCO_2_eq.

### 3.2. Uncertainties

The IPCC guideline employed in this study suggests several parameters in the model, which are believed to be very uncertain such as maximum CH_4_ producing capacity, the collection of additional industrial BOD discharged in the system. In addition the wastewater treatment pathways in the areas are assumed; however it is highly uncertain with public acceptance. Therefore, acceptance to have Johkasou system installed in household or group of households that replaced local household latrines is considered. With uncertainty level of ±10% of population acceptance to install Johkasou instead of conventional household latrines, total GHG emissions will range from ±2% (scenarios 2 and 3) to 15–22% (scenario 4).

### 3.3. Investment Requirement

The investment aspect of various wastewater treatment system proposed was analyzed based on the investment per year for the 30-year lifetime. Estimated investment for various wastewater treatment system is presented in Table 6.

**Figure 10:**
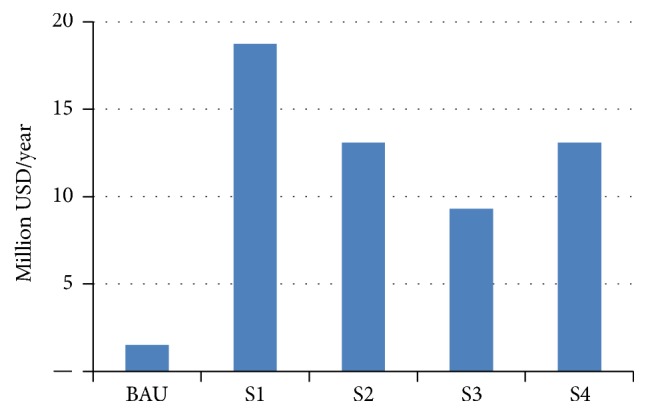
Investment for wastewater treatment system.

The result shows that the baseline scenario requires smallest investment whereas scenario 1 with centralized wastewater treatment requires the largest investment which is 1.5 million and 18.7 million US dollars per year, respectively (Figure 10). The combination system comprises decentralized system (Johkasou and latrines) in the upper streams and centralized system (sewer and wastewater treatment plant) in the populated down streams of the Vu Gia Thu Bon river basin which can reduce investment cost to 9–13 million USD per year.

### 3.4. Discussions

Methane emission from domestic wastewater increased annually from 2012 to 2040. By the year 2041 total methane emissions were 2.76 mil.tCO_2_eq and contribute 2.7% to total GHG emissions per person per year. According to simulation scenarios and economic analysis, the total GHG emissions can be reduced up to 16% compared to the baseline scenario for 30 years of project period. The increasing of CH_4_ emissions reduction resulting from the treatment of domestic wastewater was found in a study by Ma et al. [7], where the emissions reduction was 7.5%. Also if the sludge removal from wastewater treatment was included in the scope of study, methane emissions reduction would increase. In particular, sludge from Johkasou system shall be collected and treated under proper sludge management technologies such as fertilizer for urban greening or manufacturing of building materials [9].

Scenario 4 targeting 30% of rural household installs Johkasou especially in the mountainous areas in the upper and middle stream of the Vu Gia Thu Bon river basin, where the population density is relatively lower than the downstream. In populated areas downstream, centralized, or semicentralized wastewater treatment system is favored and more efficient. However, reducing the cost for installation and maintenance was seen as the important factor to aim at large scale deployment of Johkasou. Because the initial investment puts burden on the household since the cost for installation is usually 10 to 100 times higher than the cost for latrines or septic tanks.

The first Johkasou treatment unit was introduced to Vietnam by the Kubota Corporation in 2007; up to date the number of projects using Johkasou is over 1000 projects, mostly for domestic wastewater treatment at public entities (district level hospitals). Regarding the small size Johkasou for household wastewater treatment, there are few projects except for some feasibility studies, because of the high installation costs. With this development process, despite of the advanced technology of Johkasou, it is hardly implemented in the large scale. The similar issue of large scale deployment of Johkasou in developing country like Vietnam was discussed in the report from JICA [31]. To achieve Johkasou commercialization in the region, it is necessary to reduce the price for Johkasou system and meanwhile establish the carbon offsetting scheme. In particular, the emission offsetting from large scale installation of Johkasou for the region would reduce the initial cost. The target of 30% of household installing Johkasou in the Vu Gia Thu Bon river basin would likely be achieved in the condition of receiving financial support from government funding, ODA, international climate crediting schemes, and other international organization and private entities.

## 4. Conclusions

GHG emissions reduction can be achieved through treatment of domestic wastewater to achieve low-carbon watershed. Compared with baseline scenario, a maximum of 16% of total GHG emissions can be reduced in scenario 4, in which Johkasou replacing 30% of household latrines and 20% of domestic wastewater is treated by conventional activated sludge based on the projection. Emissions reduction from wastewater treatment can be credited to existing carbon-trading scheme, in order to minimize the initial cost of system construction including installation of Johkasou. On the other hand, GHG emissions can also be reduced by utilizing renewable energy for wastewater treatment which eliminates grid emissions. High uncertainty of emission calculation can be minimized by accessibility of local data or existing empirical data.

The research finding indicates that government's GHG emissions reduction target in the waste sector can be set up to 16%. Moreover, a method to develop emission inventory for wastewater treatment in rural areas of developing countries from the watershed approach is proposed. In addition, this study raises the potential of utilizing existing carbon emission trading schemes for initial investment of the wastewater treatment facilities through carbon credit.

## Figures and Tables

**Table 1:** Socioeconomic indicator of Quang Nam province, 2012.

Socioeconomic indicators	Unit	Quang Nam^1^	National^2^
GDP per capita at current price	USD	1,248	1,749
Average monthly income per capita at current price	USD	73	93
Household living under poverty line	%	17.93	14.2
Literacy rate	%	—	97.1
Household access to electricity	%	95.57	97.6
Household access to hygienic latrine	%	80.0	77.4
Urban population	%	19.18	31.84
Rural population	%	80.92	68.16

^1^[19].

^2^[20].

**Table 2:** Johkasou system wastewater treatment performance (source: [21, 22]).

Parameters	Influent	Effluent	Vietnam standard for domestic effluent [23]
COD, mg/L	200–400	22–50	—
BOD, mg/L	150–200	11–20	30
TSS, mg/L	80–200	1.6–50	50
NH_4_-N, mg/L	20–40	2–5	5
T-P, mg/L	6–8	4–6	6
Coliform, MPN/100 mL	0.1 × 10^6^–2 × 10^9^	2900–3000	3000

**Table 3:** Scenarios description and model input parameters.

Parameters	Baseline	Scenario 1	Scenario 2	Scenario 3	Scenario 4
Population increasing rate, %	0.5	0.5	0.5	0.5	0.5
BOD_5_, g/person/day	40~60	40~60	40~60	40~60	40~60
Wastewater discharge pathways, *T*_*k*,*j*_%					
(i) Latrines	80	20	20	20	20
(ii) Sewer	—	10	10	10	10
(iii) Conventional activated sludge	—	50	20	—	20
(iv) Johkasou	—	—	30	50	30
(v) Others	20	20	20	20	20
Energy consumption for CAS [24], kWh/person/year	30	30	30	30	30
Energy consumption for Johkasou, kWh/person/year	88	88	88	75	75

**Table 4:** GHG emissions projection from 10 most populated watersheds.

ID	Residential area, km^2^	Population 2041		CH_4_ emissions from wastewater, tCO_2_eq				
		People	People/km^2^	Baseline	S1	S2	S3	S4
1	9.95	79,620	8,003	336,508	258,197	270,273	317,945	269,425
2	14.26	73,418	5,149	310,295	238,084	249,220	269,721	248,438
3	5.12	26,389	5,149	111,533	85,577	89,580	96,949	89,299
4	8.54	23,885	2,798	100,949	77,456	86,943	87,749	85,716
5	10.70	22,013	2,057	93,461	71,361	80,104	80,847	78,973
6	25.09	19,426	774	79,625	63,773	68,233	68,888	67,236
7	7.69	15,813	2,057	67,488	51,219	57,500	58,034	56,688
8	28.85	15,067	522	62,165	49,870	53,329	53,837	52,556
9	7.24	14,895	2,057	61,370	49,216	52,636	53,139	51,871
10	4.10	14,668	3,578	61,994	47,567	53,393	53,888	52,639

**Table 5:** Total GHG emissions from 103 watersheds.

Parameters	Baseline	S1	S2	S3	S4
Total emission, mil tCO_2_eq	2.76	2.17	2.33	2.42	2.31
Emission reductions, mil tCO_2_eq	—	0.59	0.42	0.33	0.45
Emission reductions, %	—	21	15	12	16
Emission per capita per year, tCO_2_eq	0.15	0.11	0.12	0.13	0.12

**Table 6:** Investment (capital expenditure, CAPEX, and O&M, OPEX) for the various wastewater treatment system.

Type	CAPEX	OPEX	Note
	USD/person/year		
Latrines	3	0	Common pour-flush latrine type
Sewer	22	5	Source: Hydroconseil, 2013 [25] Estimated sewer CAPEX equals 1.6 times wastewater treatment plant CAPEX
Conventional activated sludge (CAS)	14	12	Source: [3] 1 USD = 22,500 VND (2015) Wastewater discharge 150 litres/capita/d Value for small scale wastewater treatment plant with CAS (A2O) Capacity < 10,000 m^3^/d
Johkasou	17	6	Source: [26]. Domestic Johkasou system for 5 persons
